# Relevance and limitations of clinical follow-up in a pharmacokinetic study on direct oral anticoagulants

**DOI:** 10.3389/fphar.2026.1761151

**Published:** 2026-03-02

**Authors:** Jean Terrier, Pauline Gosselin, Christophe Combescure, Pierre Fontana, Youssef Daali, Jean-Luc Reny

**Affiliations:** 1 Division of General Internal Medicine, Geneva University Hospitals, Geneva, Switzerland; 2 Division of Clinical Pharmacology and Toxicology, Geneva University Hospitals, Geneva, Switzerland; 3 Geneva Platelet Group, Faculty of Medicine, University of Geneva, Geneva, Switzerland; 4 Center of Clinical Research, Geneva University Hospital, Geneva, Switzerland; 5 Division of Angiology and Hemostasis, Geneva University Hospitals, Geneva, Switzerland

**Keywords:** apixaban, bleeding, direct oral anticoagulants, ischemic events, pharmacokinetics, plasma concentrations, real-world study, rivaroxaban

## Abstract

**Introduction:**

Direct oral anticoagulants (DOACs), including apixaban and rivaroxaban, are widely prescribed for the prevention of cardioembolic stroke and the treatment of venous thromboembolism. Although routine therapeutic monitoring is not required, real-world patients often present with multiple comorbidities or drug–drug interactions that may alter drug exposure. Observational data indicate that high DOAC plasma concentrations may increase bleeding risk, whereas low concentrations may predispose individuals to thromboembolic or ischemic events. In this study, we aimed to analyze the relationship between DOAC plasma concentrations and clinical outcomes in hospitalized patients from the OptimAT cohort.

**Methods:**

This prospective study included 200 inpatients from Geneva University Hospitals receiving apixaban (n = 100) or rivaroxaban (n = 100). Pharmacokinetic parameters (AUC 0 h–8 h, C-max, and C-trough) were derived from plasma concentrations measured using validated LC–MS/MS quantification of dried blood spot samples. Clinical follow-up was performed for 2 years to record thromboembolic, ischemic, and bleeding events, which were classified according to the ISTH criteria. Patients were categorized into percentile-based exposure groups, defined separately for apixaban and rivaroxaban. Kaplan–Meier survival curves and Cox regression models were used to assess associations between extreme versus moderate plasma concentrations and clinical outcomes.

**Results:**

During the 2-year follow-up, 27 clinical events were recorded, corresponding to an incidence rate of 9.3 per 100 patient–years for any bleeding event (95% CI: 5.57–14.44) and 3.9 per 100 patient–years for ischemic or thromboembolic events (95% CI: 1.68–7.67). These included one major bleeding event (0.5%), eighteen minor bleeding events (9%), six arterial ischemic events (3%), and two venous thromboembolic events (1%). No significant association was found between high apixaban/rivaroxaban plasma concentrations and bleeding events or between low apixaban/rivaroxaban plasma concentrations and ischemic/thromboembolic events for any pharmacokinetic parameter. The study power was limited by the small sample size and a high rate of apixaban and rivaroxaban treatment modifications or discontinuations (41%), which reduced the number of evaluable patients.

**Conclusion:**

In this real-world cohort, apixaban and rivaroxaban plasma concentrations were not associated with clinical outcomes. While highlighting the limitations of analyzing clinical outcomes within the framework of a pharmacokinetic study, these data are informative for future meta-analyses aimed at minimizing publication bias from neutral results.

## Graphical Abstract

**Figure:**
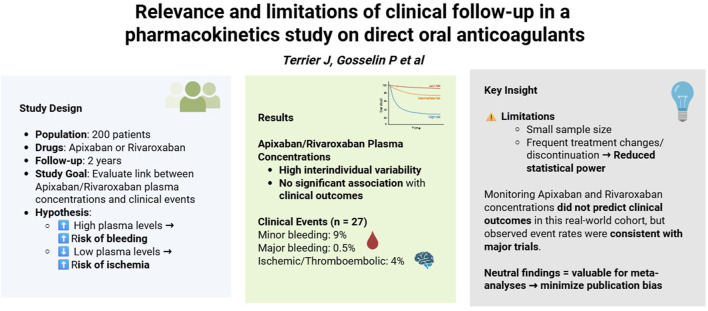

## Introduction

Direct oral anticoagulants (DOACs) are the treatment of choice for the prevention of cardioembolic stroke and the treatment of venous thromboembolism (Steffel et al., 2021; Stevens et al., 2021). Compared with vitamin K antagonists, they offer the advantage of not requiring therapeutic monitoring due to their relatively wide therapeutic range (Rahmat and Lip, 2015). However, outside of pivotal trials, which typically exclude patients with multiple comorbidities and those with significant drug–drug interactions, real-life patients may accumulate several risk factors that increase the risk of under- or overdosing (Terrier et al., 2022). Although no therapeutic interval has yet been validated (Moner-Banet et al., 2020), a growing body of evidence from both pivotal and real-life studies suggests an association between DOAC plasma concentrations and clinical outcomes (Eikelboom et al., 2017; Testa et al., 2024; Lin et al., 2023; Suwa et al., 2023; Wu et al., 2025; Ain et al., 2024). High peak (C-max) and trough (C-trough) concentrations of DOACs have been linked to an increased risk of bleeding, while low C-trough levels have been associated with ischemic events (Eikelboom et al., 2017; Testa et al., 2024; Lin et al., 2023; Suwa et al., 2023; Wu et al., 2025). A therapeutic range, thus, appears to be emerging and could be useful in guiding treatment for patients at higher risk of clinical events. In this study, we present the results of clinical follow-up of patients enrolled in the OptimAT study (NCT03477331), whose primary objective was to validate pharmacokinetic (PK) models in a hospitalized population treated with antithrombotic agents. The present analysis represents a secondary, exploratory, and hypothesis-generating endpoint of a PK-focused study aiming to describe the association between apixaban or rivaroxaban plasma concentrations and clinical events in this cohort over a 2-year prospective follow-up. Accordingly, this analysis was not designed to support causal or outcome-based inference.

## Methods

### Selection and recruitment procedures

The study design, selection, and recruitment procedures of the OptimAT prospective cohort study have been described previously (Terrier et al., 2023). In brief, clinical data were collected from inpatients at Geneva University Hospitals (HUG) who were receiving apixaban (n = 100) or rivaroxaban (n = 100). These patients were enrolled between January 2018 and June 2020. The study protocol was approved by the regional research ethics committee of the Canton of Geneva (CCER no. 2017-00225). The primary objective of the OptimAT study was to validate PK models for antithrombotic therapy, and the trial is registered in the U.S. National Institutes of Health Clinical Trials Registry (ClinicalTrials.gov, NCT03477331, registration date: 2018-03-26). Written informed consent was obtained from all participants prior to the initiation of any study procedures. The trial was conducted in accordance with the principles of the Declaration of Helsinki and the International Conference on Harmonization Good Clinical Practice (ICH-GCP) guidelines.

### Clinical follow-up

As part of the secondary endpoints of the OptimAT study, clinical events were monitored over a pre-specified 2-year follow-up. The predefined outcomes were all thromboembolic, ischemic, or bleeding complications, including the occurrence of acute venous thromboembolism (VTE) or pulmonary embolism (PE), ischemic cerebral vascular events (stroke and transient ischemic attack (TIA)), acute ischemia of a limb or organ, acute myocardial infarction, and major and clinically relevant minor bleeding according to the definition of the International Society on Thrombosis and Hemostasis (ISTH) (Schulman et al., 2005). Patients were followed up every 6 months by telephone call from the inclusion date to the time of any of the following events occurring first: discontinuation, dose modification of the primary DOAC treatment, occurrence of any of the clinical outcomes described above, death, loss to follow-up, patient withdrawal, or end of study (2 years post-inclusion). Specific questions were asked regarding cardiovascular symptoms, the occurrence of bleeding or hematomas, hospitalizations for any reason, and adherence to apixaban or rivaroxaban treatment. Patients were considered lost to follow-up if they could not be reached after six phone calls made on different days and if their treating physician had not seen them for 6 months. Data were censored at the date of the last available information. Causes of death, along with any major adverse bleeding, ischemic, or thromboembolic events, were objectively determined based on medical records.

### Observed concentrations and pharmacokinetic parameters

To ensure that the concentrations obtained were at steady state, patients had to receive the same dose of a DOAC—either rivaroxaban or apixaban—for at least 72 h. At inclusion, the dosages were as follows: apixaban—5 mg b.i.d. (n = 56), 2.5 mg b.i.d. (n = 40), and 10 mg b.i.d. (n = 4); rivaroxaban—10 mg q.d. (n = 4), 15 mg q.d. (n = 14), 20 mg q.d. (n = 59), and 15 mg b.i.d. (n = 23) and 10 mg q.d. (Terrier et al., 2022), 15 mg q.d. (Foerster et al., 2018), 20 mg q.d. (59), and 15 mg b.i.d. (23) for rivaroxaban (number of patients). Capillary blood samples were collected from patients using dried blood spots (DBSs) to obtain the observed DOAC PK parameters at 0, 0.5, 1, 2, 3, 4, 6, and 8 h post-dosage. Apixaban and rivaroxaban concentrations in blood were determined using a previously described and fully validated liquid chromatography–tandem mass spectrometry method (Terrier et al., 2023) and were converted to plasma concentrations using the DBS–plasma relationship reported by Foerster et al. (2018). PK parameters (area under the curve (AUC) 0 h–8 h using the log-trapezoidal rule), C-trough, and C-max were calculated by non-compartmental analysis using PKanalix, Luxoft, version 21 (Antony, France).

### Statistical analysis

Clinical event-free survival (hemorrhagic, ischemic, or thromboembolic) was analyzed according to apixaban and rivaroxaban quartiles of plasma AUC 0 h–8 h, C-trough, and C-max. The lowest quartile (<25th percentile) was compared with the higher quartiles (≥25th percentile) for ischemic and thromboembolic events, while the highest quartile (>75th percentile) was compared with the lower quartiles (≤75th percentile) for hemorrhagic events. Quartiles were defined separately for apixaban and rivaroxaban, given their different dosing regimens. This percentile-based approach was selected to allow the aggregation of apixaban and rivaroxaban data within a unified analytical framework while preserving sufficient group sizes for comparison. Absolute exposure thresholds previously proposed in the literature (Testa et al., 2024; Lin et al., 2023) were explored but proved unsuitable for the present cohort, as only 1% of patients exhibited C-trough concentrations below the lower threshold and 0.5% above the upper threshold, precluding meaningful statistical analysis. In addition, the objective in this study was to evaluate the relationship between plasma concentrations and clinical events for apixaban or rivaroxaban, irrespective of the indication or dosage. The use of internal percentile-based thresholds, therefore, represented a pragmatic approach consistent with the exploratory, hypothesis-generating nature of this secondary analysis. The expected clinical event rates were 1%–2% for thrombotic events and 2%–3% for bleeding events, according to pivotal studies (Ntaios et al., 2012). Kaplan–Meier survival curves were plotted to estimate the cumulative incidence of clinical events.

Hazard ratios for the association between extreme percentiles of apixaban and rivaroxaban exposure and clinical events were assessed using Cox regression models, and their two-sided 95% confidence intervals were reported. Due to the expected low rate of clinical events, the only adjustment factor for hemorrhagic events was the HAS-BLED score, which has the advantage of summarizing several factors that influence bleeding risk, namely, hypertension, renal and liver disease, history of stroke, prior major bleeding or a predisposition to bleeding, labile INR, age >65, use of medications that increase bleeding risk, and alcohol use (Pisters et al., 2010). Due to the low rate of ischemic events, no adjustment was performed for this outcome. These statistical analyses were conducted using R software, version 3.3.2 (R Foundation for Statistical Computing, Vienna, Austria). All statistical tests were two-sided with a significance level of 0.05. Since the sample size of the OptimAT study was calculated for another purpose (to validate PK models), we calculated the power to detect an association between apixaban or rivaroxaban exposure and clinical events using the fixed sample size (100 patients in each of the compared exposure group). We assumed a proportion of 55% censored data and a two-sided risk alpha of 0.05. Power was calculated using exponential survival models.

## Results

**FIGURE 1:**
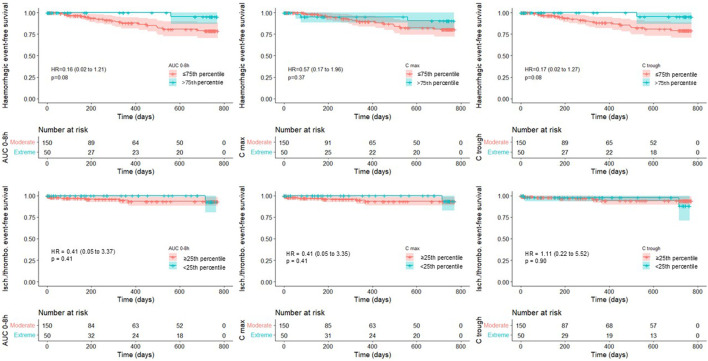
Kaplan–Meier curves showing the cumulative incidence of bleeding, ischemic, and thromboembolic events according to apixaban and rivaroxaban pharmacokinetic parameters (AUC 0 h–8 h, C-trough, and C-max). Patients in the highest quartile (>75th percentile) were compared with those in the lower quartiles (≤75th percentile) for the incidence of hemorrhagic events; patients in the lowest quartile (<25th percentile) were compared with those in the higher quartiles (≥25th percentile) for the incidence of ischemic/thromboembolic events.

A total of 100 apixaban- and 100 rivaroxaban-treated patients hospitalized in the Geneva University Hospitals were included in this study. The mean age of the population was 74.7 years, with 39% of the population being women, and the main reasons for apixaban or rivaroxaban treatment were atrial fibrillation (AF) in 76.5% vs. VTE in 23.5% of the cases (Table 1). An apixaban- or rivaroxaban-based dose consistent with the Summary of Product Characteristics (SmPC) was observed in 76% and 87% of the patients treated with apixaban and rivaroxaban, respectively. Among patients whose dose was not consistent with the SmPC, 23 of 24 (96%) patients receiving apixaban were prescribed a lower dose than recommended, compared with 7 of 13 (54%) patients treated with rivaroxaban. As shown in Supplementary Table S1, apixaban and rivaroxaban plasma concentrations exhibited wide distributions among patients. For apixaban, coefficients of variation were 38.8% for AUC 0 h–8 h, 38.8% for C-max, and 43.1% for C-trough concentrations. For rivaroxaban, coefficients of variation were 33.8% for AUC 0 h–8 h, 32.7% for C-max, and 55.1% for C-trough concentrations. Interquartile ranges and the minimum–maximum values for all pharmacokinetic parameters are reported in Supplementary Table S1. Reasons for loss to follow-up in patients without clinical events are provided in Supplementary Table S2. During the 2-year follow-up, 71 patients had their data censored due to treatment modifications (dosage changes or changes in treatment), 23 due to discontinuation of anticoagulation, 18 died (with only 1 death related to a thrombotic complication), and 2 were lost to follow-up or withdrew from the study. Changes to another oral anticoagulant, to a different apixaban or rivaroxaban dosing or discontinuation of anticoagulation occurred for the following reasons: as planned for 27 patients according to VTE treatment guidelines, arbitrarily by a treating physician for 15 patients, due to renal or hepatic failure for 13 patients, and for other reasons for 38 patients. Respectively, 23% and 28% of patients had been treated with apixaban and rivaroxaban for more than 1 week at the time of inclusion. The mean follow-up duration was 397 (standard deviation (SD) = 305) days. A total of 59 patients reached the end of the study. A total of 27 clinical events were observed (Supplementary Table S3), corresponding to an incidence rate of 9.3 per 100 patient-years for any bleeding event (95% CI: 5.57–14.44) and 3.9 per 100 patient-years for ischemic or thromboembolic events (95% CI: 1.68–7.67). These included one major bleeding event recorded (apixaban) (0.5%), 18 minor bleeding events (9%) (N = 8 for apixaban, N = 10 for rivaroxaban), 6 ischemic events (3%) (N = 3 for apixaban, N = 3 for rivaroxaban), and 2 thromboembolic events (1%) (N = 1 for apixaban, N = 1 for rivaroxaban). Patient clinical events by percentile and molecule are presented in Supplementary Tables S4, S5. The Kaplan–Meier survival curves illustrating the cumulative incidence of clinical events (bleeding, ischemic, and thromboembolic) according to apixaban and rivaroxaban PK parameters are shown in Figure 1. As previously defined, for hemorrhagic events, patients in the highest quartile of plasma concentrations (>75th percentile) for AUC 0 h–8 h, C-trough, and C-max were compared with those in the lower quartiles (≤75th percentile). Conversely, for ischemic and thromboembolic events, patients in the lowest quartile (<25th percentile) were compared with those in the higher quartiles (≥25th percentile). Cox regression analyses showed no significant association between the plasma PK parameters of patients in the lowest/highest quartiles of apixaban and rivaroxaban and the occurrence of ischemic/thrombotic or bleeding events, respectively, even after adjusting for the HAS-BLED score for bleeding events (Figure 1; Table 2). Power (%) for various assumed survivals under the alternative hypothesis and the comparison between two independent groups of 100 patients (per group) can be found in Supplementary Table S6. In the present cohort, the power was 20% to show a 5% statistically significant difference, with, for example, 95% vs. 90% of event-free survivals at 2 years between two independent groups [e.g., the highest quartile of plasma concentrations (>75th percentile) vs. the lower quartiles (≤75th percentile) for bleeding events]. The present study had 90% power to detect a 20% absolute difference in the incidence of events at 2 years (Supplementary Table S6).

**TABLE 1:** Clinical and demographic characteristics of the study population.

Characteristic	Apixaban (n = 100)	Rivaroxaban (n = 100)	All (n = 200)
Age (years), mean (sd)	76.5 (10.0)	72.8 (11.2)	74.7 (10.8)
Weight (kg), mean (sd)	77.4 (17.8)	82.1 (17.9)	79.7 (18.0)
Body mass index (kg/m^2^), mean (sd)	27.0 (5.9)	27.7 (5.9)	27.4 (5.9)
Creatinin clearance*, mean (sd)	62.2 (24.5)	76.9 (30.0)	69.6 (28.3)
Missing data	2	2	4
Drug indication (%)	AF (89), VTE (11)	AF (64), VTE (36)	AF (153), VTE (47)
Sex	​	​	​
Male	58 (58.0%)	64 (64.0%)	122 (61.0%)
Female	42 (42.0%)	36 (36.0%)	78 (39.0%)
Aspirin	28 (28.0%)	14 (14.0%)	42 (21.0%)
HAS-BLED score			
0	11 (11.0%)	12 (12.0%)	27 (11.5%)
1	32 (32.0%)	45 (45.0%)	77 (38.5%)
2	39 (39.0%)	31 (31.0%)	70 (35.0%)
3	17 (17.0%)	11 (11.0%)	28 (14.0%)
4	1 (1.0%)	1 (1.0%)	2 (1.0%)
P-glycoprotein and/or CYP3A4 inhibitor (%)	29	25	27

Abbreviations: AF, atrial fibrillation; IQR, interquartile range; VTE, venous thromboembolism. *according to the Cockroft–Gault equation.

**TABLE 2:** COX regression analyses between the pharmacokinetic parameters of apixaban and rivaroxaban and clinical events (hemorrhagic and ischemic/thromboembolic).

Parameter	Any hemorrhagic event				Ischemic/Thromboembolic event	
	Unadjusted		Adjusted		Unadjusted	
	HR (95% CI)	p	HR (95% CI)	p	HR (95% CI)	p
AUC 0 h–8 h						
≤75th percentile or ≥25th percentile^a^	1 (ref.)	​	1 (ref.)	​	1 (ref.)	​
>75th percentile or <25th percentile^a^	0.16 (0.02–1.22)	0.08	0.16 (0.02–1.21)	0.08	0.41 (0.05–3.37)	0.41
HAS-BLED score						
Per point	​	​	1.07 (0.64–1.78)	0.80	​	​
C-max						
≤75th percentile or ≥25th percentile^a^	1 (ref.)	​	1 (ref.)	​	1 (ref.)	​
>75th percentile or <25th percentile^a^	0.57 (0.17–1.96)	0.37	0.57 (0.17–1.96)	0.37	0.41 (0.05–3.35)	0.41
HAS-BLED score						
Per point	​	​	1.03 (0.61–1.72)	0.91	​	​
C-trough						
≤75th percentile or ≥25th percentile^a^	1 (ref.)	​	1 (ref.)	​	1 (ref.)	​
>75th percentile or <25th percentile^a^	0.17 (0.02–1.29)	0.09	0.17 (0.02–1.27)	0.08	1.11 (0.22–5.52)	0.90
HAS-BLED score						
Per point	​	​	1.09 (0.65–1.81)	0.75	​	​

^a^ Highest quartile of PK parameters (>75th percentile) vs. the lower quartiles (≤75th percentile) for hemorrhagic events. Lowest quartile (<25th percentile) vs. higher quartiles (≥25th percentile) for ischemic/thromboembolic events.

## Discussion

Based on the OptimAT cohort of 200 patients treated by apixaban or rivaroxaban, we failed to detect a statistically significant association between the lowest/highest quartiles of plasma concentrations for three key PK parameters (AUC 0 h–8 h, C-trough, and C-max) and the occurrence of ischemic/thrombotic or bleeding events, respectively. These neutral results contrast with several recent publications showing such an association in pivotal studies and real-world settings (Eikelboom et al., 2017; Testa et al., 2024; Lin et al., 2023; Suwa et al., 2023; Palareti et al., 2024). In these latter studies, high C-trough and C-max levels of DOACs were associated with an increased risk of bleeding, while low C-trough levels were associated with thrombotic events. Different hypotheses can be proposed to explain this discrepancy. First, the sample size of the OptimAT study was calculated for the primary objective of validating PK models. As a result, with an observed rate of 4% for ischemic/thromboembolic events and a lower-than-expected rate of 0.5% for major bleeding events, the present study was underpowered to detect such an association, as shown by our power calculation (Supplementary Table S6). In comparison, the MAS study by Testa et al. (2024) involved 1,657 patients with AF treated with DOACs, displaying similar characteristics compared to the OptimAT population, and it reported a rate of 1.3% thrombotic complications and 3.1% bleeding events (1.9% major bleeding and 1.2% clinically relevant non-major bleeding). The study by Lin et al. (2023) included 859 patients and reported a rate of 1.3% per year for thrombotic events and 1.6% per year for major bleeding events. Second, extreme low and high concentrations were infrequent in our cohort compared to those reported in other studies. Using the threshold applied by Lin et al. (2023), only 1% of OptimAT patients had lower C-trough levels and 0.5% had higher C-trough levels, compared to 14.6% and 9.0%, respectively, in the Lin et al. cohort. Third, in the MAS trial (Palareti et al., 2024), patients started DOACs within a month before the DOAC concentration measurement (Palareti et al., 2024), which is a known period of higher bleeding risk. This was not the case for our patients, as approximately a quarter had already been receiving apixaban or rivaroxaban for more than 1 week at the time of inclusion. Fourth, half of the follow-ups were interrupted before 6 months for rivaroxaban and 1 year for apixaban, respectively. Approximately one-third of the interruptions were due to treatment modifications, more than half of which were justified for planned reasons, such as VTE management guidelines or renal dysfunction. In comparison, only 1.7% of patients per year had a treatment modification or discontinuation in the MAS trial, and none of the patients changed their DOAC dosage. In other studies and across various databases, the rate of DOAC discontinuation ranges from 13% to 64% (Cools et al., 2021; Sabaté et al., 2021).

Beyond this study’s lack of power, another key limitation, shared with other related studies on the same topic, is that apixaban and rivaroxaban concentrations were measured on a given day, which may not correlate with the timing of the clinical event. We cannot guarantee that the concentrations were the same at the time of the clinical event. This is especially true in the context of hospitalization, where several factors, such as drug–drug interactions and inflammation, can affect drug concentrations (Lenoir et al., 2021). Furthermore, patients were administered different dosages based on their comorbidities and therapeutic indications. The objective, however, was to evaluate the relationship between plasma concentrations and clinical events for apixaban or rivaroxaban, irrespective of the indication or dosage. Therefore, a percentile-based approach was preferred over a threshold-based approach for the analysis. Despite these limitations, our study has several strengths. The prospective design ensured the collection of high-quality data. Additionally, clinical events were verified using medical records for validation. We also chose to censor data at the date of treatment modifications, which was not the case in other studies. This ensures that clinical events were recorded only in relation to the initial apixaban or rivaroxaban dose and the initial plasma concentration.

## Conclusion

The surprisingly high rate of apixaban and rivaroxaban modification or discontinuation in our real-life setting, coupled with a relatively small sample size of a PK study, did not allow the detection of a significant risk of ischemic/thromboembolic or bleeding events associated with low or high apixaban or rivaroxaban plasma exposure, respectively. However, monitoring clinical events within the context of such a study provides valuable descriptive data on their incidence, which is comparable to that observed in landmark trials. Although underpowered to detect small differences, this study highlights the challenges of conducting long-term pharmacokinetic–clinical outcome studies in hospitalized populations and emphasizes the value of reporting such results to reduce publication bias associated with the underreporting of neutral studies in future meta-analyses.

## Data Availability

The raw data supporting the conclusions of this article will be made available by the authors, without undue reservation.

## Group members of OptimAT study team

Chantal Csajka, Frederic Gaspar, Monia Guidi, Camille Lenoir, Victoria Rollason
