# Supplementary material for: Relevance and limitations of clinical follow-up in a pharmacokinetic study on direct oral anticoagulants
Source: Front Pharmacol. 2026 Mar 2;17:1761151. doi: 10.3389/fphar.2026.1761151 (PMC12989584; doi:10.3389/fphar.2026.1761151)
Supplement: Supplementary file 1 [file Supplementaryfile1.docx]

**SUPPLEMENTARY MATERIAL**

**Relevance and limitations of clinical follow-up in a pharmacokinetics study on direct oral anticoagulants”** by Terrier J, Gosselin P *et al.*

**Table S1. Mean and median apixaban and rivaroxaban concentrations**

|  | **AUC 0-8h** | **C max** | **C trough** |
| --- | --- | --- | --- |
| **Apixaban (n=100)** |  |  |  |
| mean (sd) | 1006 (390.1) | 155.3 (60.3) | 88.4 (38.1) |
| Median [IQR] | 967.4 [715.7 to 1280.4] | 144.9 [109.9 to 196.7] | 82.5 [58.9 to 113.6] |
| [min to max] | [215.3 to 2060.9] | [36.9 to 311.7] | [19.4 to 212.4] |
| **Rivaroxaban (n=100)** |  |  |  |
| mean (sd) | 887.3 (300.2) | 160.7 (52.6) | 47 (25.9) |
| Median [IQR] | 844.5 [670.8 to 1039.4] | 150.1 [123.4 to 184.4] | 41.3 [28.4 to 57.9] |
| [min to max] | [289 to 2173.1] | [52.7 to 332.3] | [14 to 148.7] |

**Table S2. Summary of the reasons for loss to follow-up.**

|  | Apixaban (n=100) | Rivaroxaban (n=100) | All (n=200) |
| --- | --- | --- | --- |
| Reason of end of follow-up* |  |  |  |
| Death | 14 | 4 | 18 |
| Treatment modification | 31 | 40 | 71 |
| Treatment discontinuation | 10 | 13 | 23 |
| End of study | 31 | 28 | 59 |
| Withdrawal | 1 | 1 | 2 |

*****in patients without clinical events

**Table S3. Summary of the clinical events.**

|  | Apixaban (n=100) | Rivaroxaban (n=100) | All (n=200) |
| --- | --- | --- | --- |
| Clinical events* |  |  |  |
| Major + minor bleeding | 9 | 10 | 19 |
| *Major bleeding* | *1* | *0* | *1* |
| *Minor bleeding* | *8* | *10* | *18* |
| Thrombotic + Ischemic event | 4 | 4 | 8 |
| *Thrombotic* | *1* | *1* | *2* |
| *Ischemic* | *3* | *3* | *6* |
| Any clinical event | 13 | 14 | 27 |

*****Occurring before a change or discontinuation of treatment. If a patient experiences multiple clinical events, only the first event is considered

**Table S4**. Patient clinical events by percentile for apixaban

|  | **AUC0–8h** | | | | **Cmax** | | | | **Ctrough** | | | |
| --- | --- | --- | --- | --- | --- | --- | --- | --- | --- | --- | --- | --- |
| **Percentiles** | <25 | 25-50 | 50-75 | >75 | <25 | 25-50 | 50-75 | >75 | <25 | 25-50 | 50-75 | >75 |
| **Major bleeding event, n (%)** | 1 (4%) | 0 (0%) | 0 (0%) | 0 (0%) | 1 (4%) | 0 (0%) | 0 (0%) | 0 (0%) | 1 (4%) | 0 (0%) | 0 (0%) | 0 (0%) |
| **Minor bleeding event, n (%)** | 3 (12%) | 2 (8%) | 3 (12%) | 0 (0%) | 2 (8%) | 2 (8%) | 3 (12%) | 1 (4%) | 3 (12%) | 3 (12%) | 2 (8%) | 0 (0%) |
| **Thromboembolic event, n (%)** | 0 (0%) | 0 (0%) | 1 (4%) | 0 (0%) | 0 (0%) | 0 (0%) | 1 (4%) | 0 (0%) | 0 (0%) | 0 (0%) | 1 (4%) | 0 (0%) |
| **Ischemic event, n (%)** | 0 (0%) | 2 (8%) | 1 (4%) | 0 (0%) | 0 (0%) | 2 (8%) | 1 (4%) | 0 (0%) | 1 (4%) | 0 (0%) | 2 (8%) | 0 (0%) |
| **Major + minor bleeding events, n (%)** | 4 (16%) | 2 (8%) | 3 (12%) | 0 (0%) | 3 (12%) | 2 (8%) | 3 (12%) | 1 (4%) | 4 (16%) | 3 (12%) | 2 (8%) | 0 (0%) |
| **Thromboembolic + ischemic events, n (%)** | 0 (0%) | 2 (8%) | 2 (8%) | 0 (0%) | 2 (8%) | 2 (8%) | 2 (8%) | 0 (0%) | 1 (4%) | 0 (0%) | 3 (12%) | 0 (0%) |

**Table S5**. Patient clinical events by percentile for rivaroxaban

|  | **AUC0–8h** | | | | **Cmax** | | | | **Ctrough** | | | |
| --- | --- | --- | --- | --- | --- | --- | --- | --- | --- | --- | --- | --- |
| **Percentiles** | <25 | 25-50 | 50-75 | >75 | <25 | 25-50 | 50-75 | >75 | <25 | 25-50 | 50-75 | >75 |
| **Major bleeding event, n (%)** | 0 (0%) | 0 (0%) | 0 (0%) | 0 (0%) | 0 (0%) | 0 (0%) | 0 (0%) | 0 (0%) | 0 (0%) | 0 (0%) | 0 (0%) | 0 (0%) |
| **Minor bleeding event, n (%)** | 1 (4%) | 4 (16%) | 4 (16%) | 1 (4%) | 1 (4%) | 4 (16%) | 3 (12%) | 2 (8%) | 3 (12%) | 3 (12%) | 3 (12%) | 1 (4%) |
| **Thromboembolic event, n (%)** | 0 (0%) | 0 (0%) | 0 (0%) | 1 (4%) | 0 (0%) | 0 (0%) | 0 (0%) | 1 (4%) | 0 (0%) | 0 (0%) | 0 (0%) | 1 (4%) |
| **Ischemic event, n (%)** | 1 (4%) | 1 (4%) | 0 (0%) | 1 (4%) | 1 (4%) | 1 (4%) | 0 (0%) | 1 (4%) | 1 (4%) | 1 (4%) | 0 (0%) | 1 (4%) |
| **Major + minor bleeding events, n (%)** | 1 (4%) | 4 (16%) | 4 (16%) | 1 (4%) | 1 (4%) | 4 (16%) | 3 (12%) | 2 (8%) | 3 (12%) | 3 (12%) | 3 (12%) | 1 (4%) |
| **Thromboembolic + ischemic events, n (%)** | 1 (4%) | 1 (4%) | 0 (0%) | 2 (8%) | 1 (4%) | 1 (4%) | 0 (0%) | 2 (8%) | 1 (4%) | 1 (4%) | 0 (0%) | 2 (8%) |

**Table S6**. Power (%) for various assumed survivals under the alternate hypothesis and the comparison between 2 independent groups of 100 patients (per group). The alpha risk is set at 5% two-sided.

|  |  | 2-year survival in > 75 percentiles | | | | | | |
| --- | --- | --- | --- | --- | --- | --- | --- | --- |
|  |  | 0.95 | 0.90 | 0.85 | 0.80 | 0.75 | 0.70 | 0.65 |
| 2-year survival in ≤75 percentiles | 0.95 |  | 20 | 50 | 75 | 90 | 96 | 99 |
|  | 0.90 |  |  | 14 | 38 | 63 | 83 | 93 |
|  | 0.85 |  |  |  | 12 | 31 | 56 | 77 |
|  | 0.80 |  |  |  |  | 11 | 28 | 51 |
|  | 0.75 |  |  |  |  |  | 10 | 25 |
|  | 0.70 |  |  |  |  |  |  | 9 |
